# THEMES FROM CONVERSATIONS WITH MEDICAL TRAINEES ON LGBTQ OLDER ADULTS

**DOI:** 10.1093/geroni/igac059.3032

**Published:** 2022-12-20

**Authors:** Shobhana Sandhu, Mackenzi Kim, Lynn Wilson, Nyann Biery, Kimberly Infante

**Affiliations:** Lehigh Valley Health Network, Allentown, Pennsylvania, United States; Lehigh Valley Health Network, Allentown, Pennsylvania, United States; Lehigh Valley Health Network, Allentown, Pennsylvania, United States; Lehigh Valley Health Network, Allentown, Pennsylvania, United States; Lehigh Valley Health Network, Allentown, Pennsylvania, United States

## Abstract

Approximately 6–12% of the US population 65 or older self-identifies as LGBTQ. This population faces immense barriers when accessing care, including the bias from healthcare professionals. Efforts to combat this bias through formal education are minimal. Using a mixed methods study with one-group pretest-posttest design and focus groups, medical learners were included in sessions involving a showing of Gen Silent and a post-viewing discussion. Themes from discussion were extracted by two independent reviewers. Medical learners (Nf15) included residents and faculty of psychiatric and emergency medicine at Lehigh Valley Health Network. Themes included: recognition of the social isolation faced by LGBTQ older adults, recognition of barriers to care including stigma and bias, challenges supporting patients and enabling patients’ openness, a need for a community resource repository, opportunities for EMR optimization, and physicians as advocates. These results highlight the need for additional training for medical trainees as well as the efficacy of using a tool like Gen Silent to accomplish this.

